# AaEIN3 Mediates the Downregulation of Artemisinin Biosynthesis by Ethylene Signaling Through Promoting Leaf Senescence in *Artemisia annua*

**DOI:** 10.3389/fpls.2018.00413

**Published:** 2018-04-05

**Authors:** Yueli Tang, Ling Li, Tingxiang Yan, Xueqing Fu, Pu Shi, Qian Shen, Xiaofen Sun, Kexuan Tang

**Affiliations:** Joint International Research Laboratory of Metabolic & Developmental Sciences, Key Laboratory of Urban Agriculture (South) Ministry of Agriculture, Plant Biotechnology Research Center, Fudan-SJTU-Nottingham Plant Biotechnology R&D Center, School of Agriculture and Biology, Shanghai Jiao Tong University, Shanghai, China

**Keywords:** ethylene, AaEIN3, leaf senescence, downregulation, artemisinin

## Abstract

Artemisinin is an important drug for malaria treatment, which is exclusively produced in *Artemisia annua*. It’s important to dissect the regulatory mechanism of artemisinin biosynthesis by diverse plant hormones and transcription factors. Our study shows ethylene, a plant hormone which accelerates flower and leaf senescence and fruit ripening, suppressed the expression of genes encoding three key enzymes ADS, DBR2, CYP71AV1, and a positive regulator AaORA involved in artemisinin biosynthesis. Then we isolated the gene encoding ETHYLENE-INSENSITIVE3 (EIN3), a key transcription factor in ethylene signaling pathway, by screening the transcriptome and genome database from *Artemisia annua*, named *AaEIN3*. Overexpressing *AaEIN3* suppressed artemisinin biosynthesis, while repressing its expression with RNAi enhanced artemisinin biosynthesis in *Artemisia annua*, indicating AaEIN3 negatively regulates artemisinin biosynthesis. Further study showed the downregulation of artemisinin biosynthesis by ethylene required the mediation of AaEIN3. AaEIN3 could accelerate leaf senescence, and leaf senescence attenuated the expression of *ADS, DBR2, CYP71AV1*, and *AaORA* that are involved in artemisinin biosynthesis. Collectively, our study demonstrated a negative correlation between ethylene signaling and artemisinin biosynthesis, which is ascribed to AaEIN3-induced senescence process of leaves. Our work provided novel knowledge on the regulatory network of plant hormones for artemisinin metabolic pathway.

## Introduction

Malaria is a malignant infectious disease caused by plasmodium, severely threatening humans’ health. Nowadays this disease is still prevalent in many areas of Southeast Asia and Africa. Artemisinin is a well-known remedy for curing malaria, which is isolated from *Artemisia annua* and was found first by Chinese scientists in the 1970s. Artemisinin Combination Therapy (ACT) has become the first choice for curing malaria recommended by World Health Organization (WHO) (Mutabingwa, 2005; Graham et al., 2010; Weathers et al., 2011). Meanwhile, artemisinin and its derivatives have been found to have anti-schistosomiasis, anti-tumor, and anti-inflammatory activities (Bhattarai et al., 2007; Efferth, 2007; Efferth et al., 2008). Therefore, artemisinin and its derivatives have a good prospect for application as a multi-purpose natural medication. Artemisinin is mainly extracted from leaves of *Artemisia annua*, but its content in wild *Artemisia annua* is low (0.1–0.8% dry weight) (Duke et al., 1994; Kumar et al., 2004). So it’s of great significance to elevate artemisinin production in *Artemisia annua* by all kinds of strategies like metabolic engineering, environmental regulation and genetic breeding. Studying and understanding the regulatory mechanisms of artemisinin biosynthesis by diverse plant hormones and transcription factors will conduce to people’s practice for improving artemisinin production by the above strategies.

The precursor for artemisinin biosynthesis is farnesyl diphosphate (FDP) containing three isoprenyl 5-carbon (C5) units. FDP is formed by the condensation of three isoprenyl diphosphates (IPPs) through the catalysis of farnesyl diphosphate synthase (FPS). Then FDP is converted into dihydroartemisinic acid (DHAA) by sequential catalysis of amorpha-4,11-diene synthase (ADS), cytochrome P450 monooxygenase (CYP71AV1), artemisinic aldehyde Δ11 (13) reductase (DBR2) and aldehyde dehydrogenase 1 (ALDH1) (Tang et al., 2014). Finally, the conversion of DHAA into artemisinin is an automatic reaction under light, without the need of enzymatic catalysis (Sy and Brown, 2002; Brown and Sy, 2004).

Many transcription factors are found to participate in artemisinin metabolic regulation. The first discovered transcription factor that positively regulates artemisinin biosynthesis in *Artemisia annua* is AaWRKY1, which activates the expression of key enzyme genes in artemisinin biosynthetic pathway (Ma et al., 2009). Afterwards, other transcription factors, such as AaERF1 and AaERF2 (Yu et al., 2012), AaORA (Lu et al., 2013), AaMYC2 (Shen et al., 2016), AabZIP1 (Zhang et al., 2015), were successively discovered to act positive roles in the regulation of artemisinin biosynthetic pathway. Now it has been found that some plant hormones as jasmonate (JA) (Wang et al., 2010; Caretto et al., 2011), abscisic acid (ABA) (Jing et al., 2009) and salicylic acid (SA) (Pu et al., 2009; Aftab et al., 2011) could increase artemisinin production. And the regulatory mechanism of artemisinin biosynthesis by these hormones signaling has been partially revealed. The regulation of artemisinin biosynthesis by these hormones mainly involves the functioning of MYC2, bZIP1, and NAC-like transcription factors, respectively (Zhang et al., 2015; Lv et al., 2016; Shen et al., 2016). Moreover, it was reported that adding ethephon in the medium led to a decrease of artemisinin content in the roots of *Artemisia annua* seedlings (Weathers et al., 2005), but it’s still unclear about the mechanism being responsible for the decrease of artemisinin content under ethephon treatment.

Ethylene is a major plant hormone, modulating the process of plant development, secondary metabolism and stress response. For example, ethylene could cause morphological changes in plant seedlings grown in darkness, including the repression of root and hypocotyl’s extension, the thickening of hypocotyl’s lateral growth, and the exaggerated apical hook curvature (Bleecker et al., 1988; Ecker, 1995). And as a well-known senescence inducer, ethylene could accelerate fruit ripening and the senescence of flower and leaves (Abeles et al., 1988). In the regulatory process by ethylene signaling, ethylene signals are first perceived by and bind with ethylene-receptor proteins: ETR1/2, ERS1/2, and EIN4, leading to the inactivation of receptor-CTR1 complex (Kieber et al., 1993; Hua and Meyerowitz, 1998). Inactive receptor-CTR1 complex cannot phosphorylate EIN2, a component downstream in ethylene signaling pathway, so that EIN2 would not be degraded and gets activated (Alonso et al., 1999; Qiao et al., 2012). Then active EIN2 protein suppresses the accumulation of two F-box proteins EBF1 and EBF2, thus precluding the degradation of EIN3/EIL1 protein via the EBF1/2-mediated 26S ubiquitin-proteasome pathway (Guo and Ecker, 2003; An et al., 2010). So at the presence of ethylene, EIN3/EIL1 protein can be maintained and accumulate stably, which would regulate the expression of ERF transcription factors and thereby activate the expression of ethylene-responsive genes downstream (Guo, 2011). Besides that ethylene’s presence could enhance the stability of EIN3/EIL1 protein to increase its accumulation, the transcription level of *EIN3* gene in leaves would increase with leaves aging. EIN3 protein in *Arabidopsis* could directly repress microRNA164 transcription, thus promoting the expression of senescence-associated genes *SAG12* and *NAC2* (also named *ORE1*) and accelerating the process of leaf senescence. Therefore, *EIN3* is a senescence-associated gene, involved in regulating the process of leaf senescence induced by ethylene or aging (Li et al., 2013).

Previous experimental results of our lab showed that *ADS, DBR2, CYP71AV1*, three key enzyme genes in artemisinin biosynthetic pathway and *AaORA*, a positive regulator in artemisinin biosynthesis, have relatively high expression level in young leaves of *Artemisia annua*. As leaves get matured and senescent, their expression level drops rapidly (Lu et al., 2013). Meanwhile, the content of DHAA, the end-product of enzymatic reactions in artemisinin biosynthetic pathway, is relatively high in young leaves, and its content declines sharply with leaf maturation and aging (Zhang et al., 2012). This indicated that leaf aging and senescence will attenuate artemisinin biosynthesis.

This study mainly focuses on the regulatory effect of ethylene signaling pathway on artemisinin biosynthesis and tries to unravel the possible mechanism behind it. Our results demonstrates that ethylene negatively regulates the expression of *ADS, DBR2, CYP71AV1*, and *AaORA* that are involved in artemisinin biosynthesis, and that such negative regulation is associated with leaf senescence induced by EIN3, a key component acting in ethylene signaling pathway. Our work revealed a possible mechanism by which ethylene affects artemisinin biosynthesis and provided more knowledge and clues for researching the regulatory effect of plant hormones on artemisinin metabolic pathway.

## Materials and Methods

### Cloning and Homology Analysis of *AaEIN3*

By analyzing the sequence information in transcriptome database and full genome database of *Artemisia annua* established by our lab (most of sequences in the databases have been annotated), and by sequence alignment with reported EIN3/EIL1s from other plant species, the gene sequence highly homologous to other *EIN3/EIL1*s was selected out from the transcriptome database of *Artemisia annua*, named as *AaEIN3*. Homologous alignments of nucleotide and protein sequences were performed with Protein-Blast Tool at NCBI website and Vector NTI 9.0 software. Phylogenetic analysis of AaEIN3 was done through neighbor-joining (NJ) method with MEGA 5.0 software. Full-length coding region of *AaEIN3* was obtained and amplified with primers AaEIN3-ORF1 (5′-GGATCCATGGGGATGGGGATCTTTGAAG-3′) and AaEIN3-ORF2 (5′-CTGCAGTCAAAGGTACCACATTG ACATATC-3′).

### Plant Hormone Treatment

*Artemisia annua* plants were grown in soil matrix in a chamber (16 h light/8 h dark) at 25°C for the day/22°C for the night with 65% relative humidity. For analysis of gene expression mode under ethephon (Et) and aminoxyacetic acid (AOA) treatments, 500 μM Et solution, 200 μM AOA solution and sterile water (as the mock) were sprayed evenly over 14-day-old seedlings of *Artemisia annua* separately. Young leaves at the same position from 8 to 10 seedlings were excised and gathered in the microfuge tube as one biological sample after 0, 1, 3, 6, 9, 12, and 24 h of the treatment, respectively. These samples were stored at -80°C for subsequent RNA extraction and qPCR analysis. For analysis of gene expression mode in transgenic plants under Et treatment, 500 μM Et solution was sprayed evenly over *AaEIN3*-ox, WT, and RNAi plant lines. Then the 2nd leaves counted downward from top meristems were sampled after 0 and 6 h of the treatment, and stored at -80°C for subsequent RNA extraction and qPCR analysis.

### Gene Expression Analysis by qPCR

The expression levels of all genes of interest in the study were detected by quantitative PCR (qPCR). Total RNA was extracted from plant samples with the Plant RNA Extraction Kit (Tiangen Biotech, Peking, China) according to the kit’s instructions. Aliquots of 1 μg total RNA was used for cDNA synthesis in a reverse transcription system (Takara, Tokyo, Japan). The amplification reaction of qPCR was performed in a Roche LightCycler 96 Real-Time PCR Device, using SYBR Green qPCR Master Mix reagents (Tiangen Biotech, Peking, China) according to the manufacturer’s instructions. Relative expression levels of genes were normalized to the expression of β*-Actin* from *Artemisia annua*. The specific primers for each gene used in qPCR are listed in Supplementary Table S1. mRNA expression levels of the target genes were measured with 2^-ΔC_t_^.

### Vector Construction and Transformation of *Artemisia annua*

For *AaEIN3*-overexpression vector construction, the coding region of *AaEIN3* with a BamHI and a PstI restriction site on either end, respectively, was ligated into the pHB+ vector under the control of the CaMV35S promoter to generate pHB-35S:sGFP-AaEIN3:Noster construct, with the sGFP fused to the N-terminal of AaEIN3. For RNA interference (RNAi) vector construction, the primers AaEIN3-RiF (5′-CACCTGAATCGTGGCGGAACGCTAAA-3′) and AaEIN3-RiR (5′-ACTGAAACCCTGCTGGCATAAA-3′) were designed to amplify a 350 bp-long RNAi fragment with cDNA of *AaEIN3* as the template. By using the gateway cloning system, the RNAi fragment was first ligated into TOPO vector and then cloned into the pHellsGate12 vector via LR reaction (Invitrogen, United States) to generate the final pHellsGate-RNAi construct. The resulting *AaEIN3*-overexpression and RNAi constructs were transduced into *Agrobacterium tumefaciens* strain EHA105, respectively, and then introduced into *Artemisia annua* to generate transgenic *Artemisia annua* plants as previously described (Shen et al., 2012). Independent transgenic lines were grown and selected in hygromycin-containing MS medium for pHB-*AaEIN3* overexpression transformants, and in kanamycin-containing MS medium for pHellsGate-RNAi transformants. *AaEIN3*-overexpression plant lines were confirmed by PCR detection with primers AaEIN3-ORF1 (5′-GGATCCATGGGGATGGGGATCTTTGAAG-3′) and rbc48-A (5′-GCATTGAACTTGACGAACGTTGTCGA-3′). And *AaEIN3*-RNAi lines were confirmed by PCR detection with primers p35S-FP (5′-TTCGTCAACATGGTGGAGCA-3′) and AaEIN3-RiR (5′-ACTGAAACCCTGCTGGCATAAA-3′). These transgenic lines were transferred to soil and kept for further analyses.

### HPLC Analysis of Dihydroartemisinic Acid (DHAA) and Artemisinin Contents

HPLC analysis was used to detect the contents of DHAA and artemisinin in leaves of *Artemisia annua*. Samples were prepared as described previously (Lu et al., 2013). *Artemisia annua* leaves were dried in a drying oven at 45–50°C for 48–72 h, and then ground to powder in a mortar. 0.1 g dried leaf powder was added into 1.5 ml methanol and ultrasonically oscillated for 30 min at 25°C/50W. After centrifugation at 10000*g* for 10 min, the clear supernatant was collected and the extraction was repeated once more. The resulting supernatant was filtered through a 0.22-μm membrane. The filtrates were analyzed by a Waters Alliance 2695 HPLC system coupled with a Waters 2420 ELSD detector (Milford, MA, United States). The mobile phase was methanol/H_2_O (v:v = 6:4) for artemisinin measurement, and acetonitrile/0.1% acetate (v:v = 6:4) for DHAA measurement. The HPLC conditions were set as described previously (Lu et al., 2013). The standard of artemisinin was purchased from Sigma-Aldrich (Shanghai, China), and the standard of DHAA was purchased from Honsea Sunshine Bio Science & Technology Co., Ltd. (Guangzhou, China).

## Results and Discussion

### Ethylene Negatively Regulates the Expression of Genes Involved in Artemisinin Biosynthesis

**FIGURE 1:**
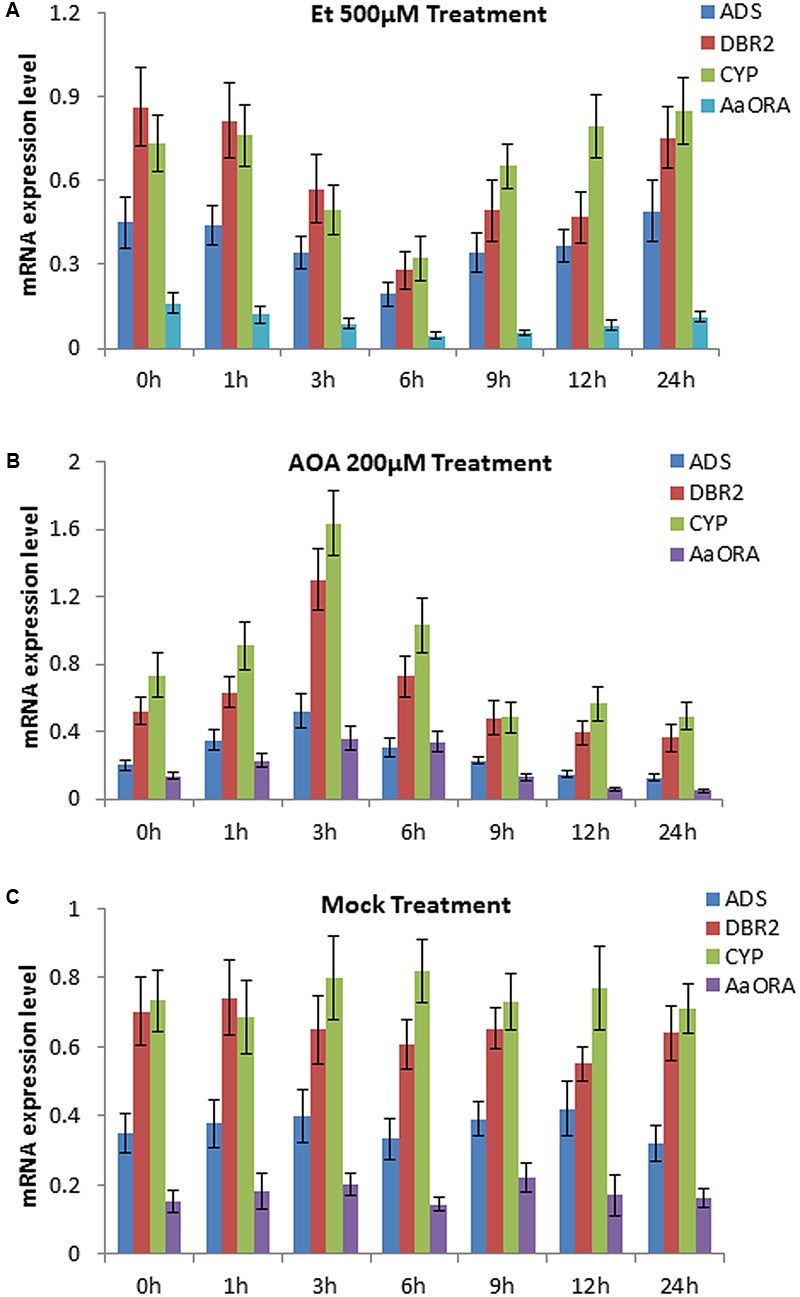
Expression levels of genes involved in artemisinin biosynthesis at different time points after ethephon (Et) and aminoxyacetic acid (AOA) treatments, measured by qPCR. Sterile water was used as the mock treatment. **(A)** Expression levels of *ADS, DBR2, CYP71AV1* (*CYP*), and *AaORA* after 0, 1, 3, 6, 9, 12, and 24 h of 500 μM Et treatment. **(B)** Expression levels of *ADS, DBR2, CYP71AV1* (*CYP*), and *AaORA* after 0, 1, 3, 6, 9, 12, and 24 h of 200 μM AOA treatment. **(C)** Expression levels of *ADS, DBR2, CYP71AV1* (*CYP*), and *AaORA* after 0, 1, 3, 6, 9, 12, and 24 h of sterile water treatment as the mock. Error bars indicate ± SD of three experimental replicates.

An earlier report showed that applying 15 mg/L ethephon (Et) in the growing medium of *Artemisia Annua* seedlings would lead to a decrease of artemisinin content in the roots (Weathers et al., 2005). To detect whether ethylene has an impact on the expression of artemisinin biosynthesis-related genes, we sprayed 500 μM Et solution and 200 μM AOA (an inhibitor of endogenous ethylene biosynthesis) respectively, to 14-day old wild type seedlings of *Artemisia annua.* The plants of mock group were treated with sterile water. qPCR was done to detect the expression mode of *ADS, DBR2, CYP71AV1* (*CYP*), and *AaORA*, four important artemisinin biosynthesis-associated genes at different time points after the treatment. The result showed that Et treatment significantly downregulated the expression level of *ADS, DBR2, CYP71AV1*, and *AaORA* in leaves, compared to the mock group (**Figures 1A,C**). The expression level of the four genes dropped to the lowest level after 6 h of Et treatment, and gradually rose back to the normal level afterward (**Figure 1A**). Meanwhile, treatment with AOA significantly upregulated the expression level of *ADS, DBR2, CYP71AV1*, and *AaORA* in leaves, compared to the mock group (**Figures 1B,C**). The expression level of the four genes rose to the peak after 3 h of AOA treatment, and gradually dropped back afterward (**Figure 1B**). This indicated that suppressing endogenous ethylene biosynthesis could enhance the expression of *ADS, DBR2, CYP71AV1*, and *AaORA*. The above result demonstrated that ethylene negatively regulates the expression of three key enzyme genes *ADS, DBR2, CYP71AV1*, and a positive regulator gene *AaORA* that are involved in artemisinin biosynthesis.

### Isolation and Characterization of EIN3 Sequence in *Artemisia Annua*

ETHYLENE-INSENSITIVE3 (EIN3) is a key transcription factor in ethylene signaling pathway. Ethylene regulates the expression of ethylene-responsive genes downstream via EIN3/EIL1’s functioning, thus further affecting diverse physiological courses of plants as germination and development, secondary metabolism and stress response. We speculated, the repression of the expression of these artemisinin biosynthesis-associated genes by ethylene may involve the mediation of EIN3/EIL1 protein. Therefore, dissecting the role of EIN3 in ethylene’s regulating artemisinin biosynthesis became our major goal in the research.

**FIGURE 2:**
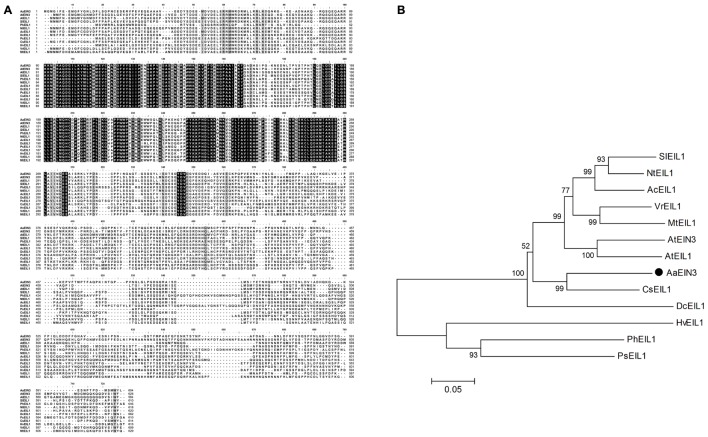
Sequence characterization of AaEIN3. **(A)** Sequence alignment of EIN3/EIL1s. Amino acid sequences were aligned as follows: AaEIN3 (*Artemisia annua*), AtEIN3 (*Arabidopsis thaliana*, Genbank Accession No. AAC49749), AtEIL1 (*Arabidopsis thaliana*, AEC07930), SlEIL1 (*Solanum lycopersicum*, AAK58857), PhEIL1 (*Petunia x hybrid*, AAR08677), NtEIL1 (*Nicotiana tabacum*, AAP03997), AcEIL1 (*Actinidia chinensis*, AID55343), DcEIL1 (*Dianthus caryophyllus*, AAF69017), PsEIL1 (*Paeonia suffruticosa*, AFI61907), CsEIL1 (*Citrus sinensis*, NP_001275851), HvEIL1 (*Hordeum vulgare*, ADO21118), VrEIL1 (*Vigna radiata*, AAL76272), and MtEIL1 (*Medicago truncatula*, ACX54782). The completely identical amino acids were shown with capital letters against black background. Less conserved amino acids were shown with capital letters against dark-gray or relatively light-gray background. Non-conserved amino acids were shown with capital letters against white background. The darker background there is, the higher sequence homology there is. **(B)** Phylogenetic analysis of EIN3/EIL1s from different plant species. AaEIN3 was marked with•.

Through homologous alignments among the sequences from transcriptome database of *Artemisia Annua* and EIN3/EIL1 gene sequences reported in other species, we isolated the gene sequence of *EIN3* from *Artemisia Annua* transcriptome database, which is highly homologous to *EIN3*/*EIL1* sequences from other species, named *AaEIN3*. *AaEIN3* encodes 604 amino acids, with a coding region of 1815 bp. Multiple alignment showed an identity of 56–67% in protein sequence between AaEIN3 and EIN3/EIL1s from other plant species (**Figure 2A**). Phylogenetic analysis showed AaEIN3 is closest in evolutionary relationship to EIL1 from *Citrus sinensis* (CsEIL1) (**Figure 2B**).

### AaEIN3 Negatively Regulates Artemisinin Biosynthesis

**FIGURE 3:**
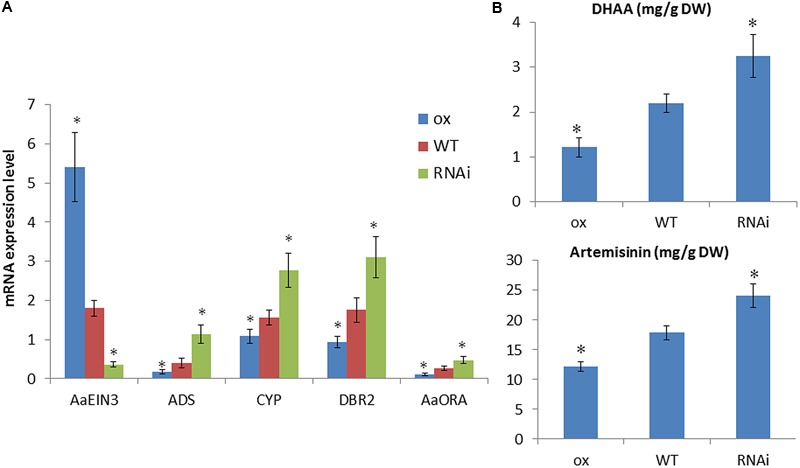
Analyses of **(A)** expression levels of *AaEIN3, ADS, DBR2, CYP71AV1* (*CYP*), and *AaORA* and **(B)** DHAA and artemisinin contents in *AaEIN3*-ox, WT, and RNAi plant lines. Data is the mean value ± SD of biological replicates (*n* ≥ 5) from different independent plant lines. Asterisk indicated a significant difference compared to the value of WT (Student’s *t*-test, *P* < 0.05).

To detect whether AaEIN3 affects artemisinin biosynthesis, we constructed *AaEIN3*-overexpressing (ox) vector and RNAi vector, respectively, and transformed them into *Artemisia Annua* plants. PCR was done to select out *AaEIN3*-overexpressing (ox) and RNAi transgenic plant lines. These transgenic plants were kept for further analysis. qPCR result showed that the expression level of *AaEIN3* in *AaEIN3*-ox plants was significantly elevated, compared to wild type plants (WT), while the expression level of *ADS, DBR2*, and *CYP71AV1*, three key enzyme genes in artemisinin biosynthesis, was significantly lower than that of WT (**Figure 3A**). Meanwhile, the expression level of *AaORA*, a transcription factor positively regulating these key enzyme genes expression, also significantly declined relative to that in WT (**Figure 3A**). This indicated that AaEIN3 overexpression reduced the expression of *ADS, DBR2, CYP71AV1*, and *AaORA*, which are involved in artemisinin biosynthesis. On the other hand, in RNAi plants, the expression of *AaEIN3* got significantly repressed, compared to that in WT, while the expression level of *ADS, DBR2, CYP71AV1*, and *AaORA* was significantly higher than that in WT (**Figure 3A**). This indicated that repression of *AaEIN3* expression led to an increase of *ADS, DBR2, CYP71AV1*, and *AaORA* expression. These results demonstrated AaEIN3 negatively regulates the expression of *ADS, DBR2, CYP71AV1*, and *AaORA* that are involved in artemisinin biosynthesis.

Then we detected the contents of DHAA and artemisinin in *AaEIN3*-ox, RNAi and WT plants by HPLC. The result showed that in *AaEIN3*-ox plants, both of DHAA and artemisinin contents were significantly lower than that in WT plants; while in RNAi plants, both of DHAA and artemisinin contents were significantly higher than that in WT plants (**Figure 3B**). These above results demonstrated AaEIN3 is a negative regulator in artemisinin biosynthesis.

### The Downregulation of Artemisinin Biosynthesis by Ethylene Requires the Mediation of AaEIN3

**FIGURE 4:**
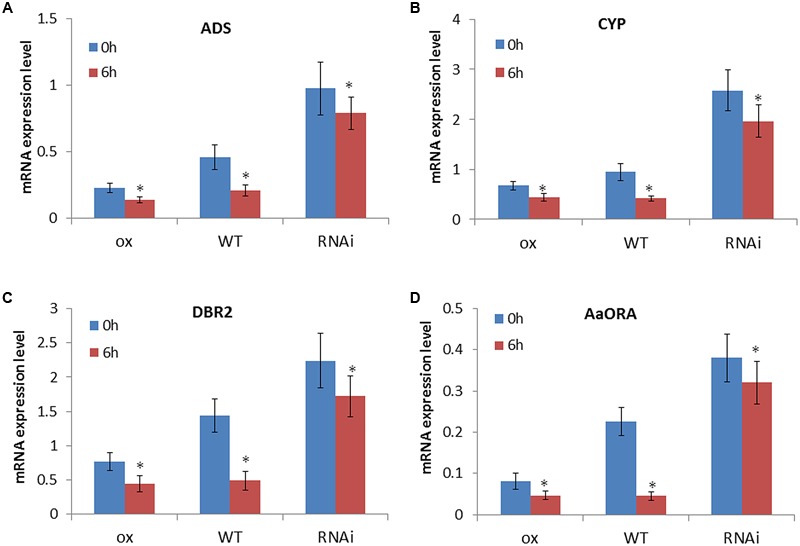
Expression levels of **(A)** *ADS*, **(B)** *CYP71AV1* (*CYP*), **(C)** *DBR2*, and **(D)** *AaORA* in *AaEIN3*-ox, WT, and RNAi plants after 0 and 6 h of 500 μM ethephon (Et) treatment. Data is the mean value ± SD of biological replicates (*n* = 5) from different independent plant lines. Asterisk indicated a significant difference compared to the value at 0 h under Et treatment (Student’s *t*-test, *P* < 0.05).

To detect whether the downregulation of artemisinin biosynthesis by ethylene involves the function of AaEIN3, we evenly sprayed *AaEIN3*-ox, WT, and RNAi plants with 500 μM Et solution, and detected the expression mode of *ADS, DBR2, CYP71AV1*, and *AaORA* in leaves of these plant lines at 0 and 6 h after the treatment. The result is shown in **Figure 4**. Et treatment led to a decline of expression of *ADS, DBR2, CYP71AV1*, and *AaORA* in all of *AaEIN3*-ox, WT, and RNAi plant lines. But in WT, the downregulation effect of Et on the four genes’ expression was the most significant of all. When *AaEIN3* expression got repressed by RNAi, the downregulation effect of Et on the expression of *ADS, DBR2, CYP71AV1*, and *AaORA* got significantly attenuated relative to that in WT, or rather the responsiveness of the four genes to ethylene signals got attenuated when *AaEIN3* expression was repressed by RNAi. These results indicated that ethylene signals downregulate artemisinin biosynthesis via the mediation of AaEIN3.

What is more, overexpression of *AaEIN3* significantly reduced the expression level of *ADS, DBR2, CYP71AV1*, and *AaORA*, and Et treatment further attenuated the four genes’ expression in *AaEIN3*-ox plants. But in *AaEIN3*-ox plants, the extent of the reduction in the four genes’ expression level was not so significant as that in WT after Et treatment (**Figure 4**). We speculate the expression amount of AaEIN3 in *AaEIN3*-ox plants has surpassed the normal need for regulating the genes downstream, and is sufficient to downregulate the expression level of those genes to the full extent. Ethylene could repress the degradation of EIN3 protein to make it accumulate gradually. Under the context of *AaEIN3* overexpression driven by 35S promoter, the further accumulation of EIN3 impelled by exogenous Et strengthened the negative regulation effect of EIN3 on genes expression not so significantly as that in WT. This may explain why the decline in the four genes’ expression level in *AaEIN3*-ox plants after Et treatment is much lesser than that in WT plants. This result also suggested a crucial role of AaEIN3 in the regulation of artemisinin biosynthesis by ethylene signaling pathway from the other side.

### The Downregulation of Artemisinin Biosynthesis by Ethylene Is Associated With AaEIN3-Induced Leaf Senescence

Previous study of our lab found that the expression level of *ADS, DBR2, CYP71AV1*, and *AaORA*, and the content of DHAA, the end-product of enzymatic reactions in artemisinin biosynthesis, are relatively higher in younger leaves. As leaves get matured and senescent, the four genes expression level and DHAA content get lower rapidly, that demonstrated that the process of leaf maturation and senescence repressed the expression of these genes participating in artemisinin biosynthesis (Zhang et al., 2012; Lu et al., 2013). Besides, an earlier study reported that EIN3 transcription in leaves of *Arabidopsis* would increase with leaves aging, and that EIN3 protein could directly repress miR164 transcription to increase the expression of senescence-associated genes as *NAC2* (also named *ORE1*) and *SAG12*, thus accelerating leaf senescence (Li et al., 2013). Therefore, *EIN3* is a senescence-associated gene, which can promote the process of ethylene- or age-dependent leaf senescence. Based on the previous findings, we infer that ethylene’s negative regulation of artemisinin biosynthesis may correlate with EIN3-induced leaf senescence.

**FIGURE 5:**
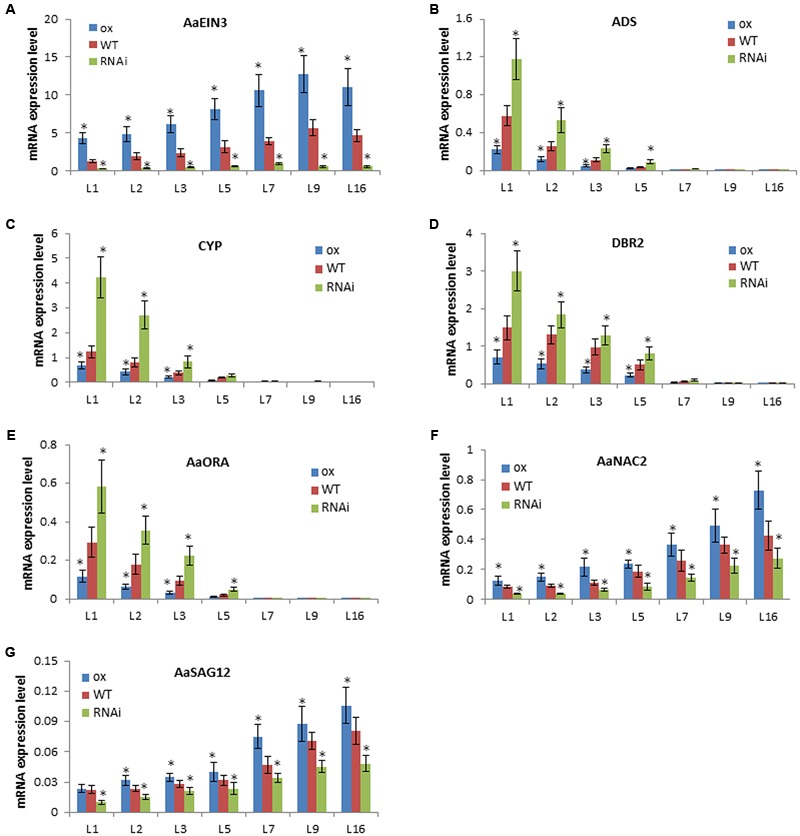
Gene expression levels in leaves at different developmental stages in *AaEIN3*-ox, WT, and RNAi plant lines. **(A–G)** show the expression level of *AaEIN3, ADS, CYP71AV1* (*CYP*), *DBR2, AaORA, AaNAC2*, and *AaSAG12*, respectively, in the 1st, 2nd, 3rd, 5th, 7th, 9th, 16th leaf (L1, L2, L3, L5, L7, L9, L16) counted downward from the top meristem in *AaEIN3*-ox, WT, and RNAi plants. The lower located, the more aged and senescent the leaf is. Data is the mean value ± SD of biological replicates (*n* = 5) from different independent plant lines. Asterisk indicated a significant difference compared to the value of WT (Student’s *t*-test, *P* < 0.05).

To confirm such inference, qPCR analysis was done to detect the expression mode of genes in leaves at different ages or developmental stages in the same phyllotaxy. On a stem of the plant, the closer to top meristems, the younger the leaf is; the lower located, the more aged and senescent the leaf is. qPCR result showed that *AaEIN3* expression level is lowest in the youngest leaf closest to top meristems (marked as L1 in **Figure 5**), and gets higher as leaf becomes aged and senescent in all of *AaEIN3*-ox, WT, and RNAi plant lines (**Figure 5A**). But the expression mode of *ADS, CYP71AV1, DBR2*, and *AaORA*, is on the contrary to that of *AaEIN3*: the more aged and senescent the leaf is, the lower their expression level gets (**Figures 5B–E**). This result is consistent with the previous report, indicating the aging and senescence of leaves attenuated the expression of the four genes involved in artemisinin biosynthesis.

Then, to detect whether AaEIN3 expression could accelerate leaves’ senescence process, we screened *Artemisia Annua* transcriptome database and its full genome database (the sequence information of both databases has not been published yet), and selected out the cDNA sequences of a senescence-associated gene *NAC2* and a senescence marker gene *SAG12* in *Artemisia Annua*, named *AaNAC2* and *AaSAG12*, respectively. qPCR result showed that the expression level of *AaNAC2* and *AaSAG12* increased with the increase of leaves age (**Figures 5F,G**), as does the expression level of *NAC2* (*ORE1*) and *SAG12* in *Arabidopsis thaliana* reported previously (Li et al., 2013). Through comparing the expression level of *AaNAC2* and *AaSAG12* in the leaves at the same developmental stage (ranking at the same position in the phyllotaxy) among *AaEIN3*-ox, WT, and RNAi plants, we found *AaEIN3* overexpression increased *AaNAC2* and *AaSAG12* expression, while repressing *AaEIN3* expression by RNAi reduced *AaNAC2* and *AaSAG12* expression (**Figures 5F,G**). This indicated *AaEIN3* could promote the expression of leaf senescence-associated genes.

**FIGURE 6:**
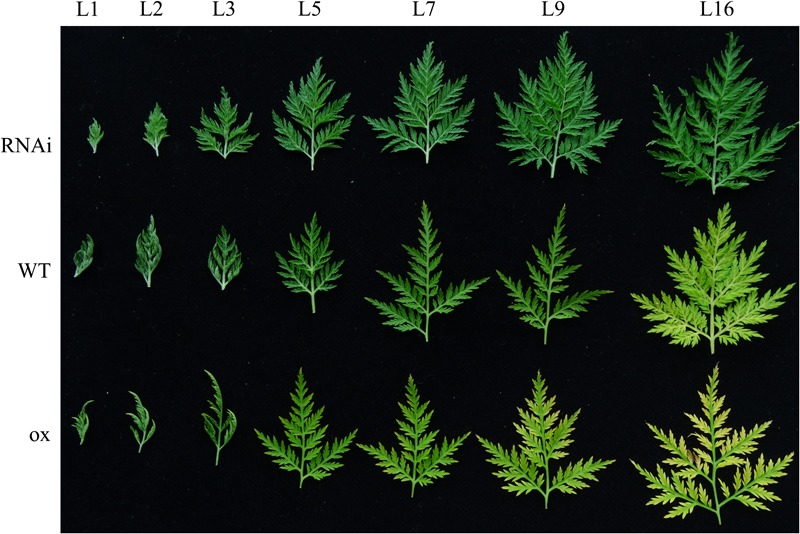
The phenotype difference of leaves at different developmental stages on the phyllotaxy among *AaEIN3*-ox, WT, and RNAi plants. L1, L2, L3, L5, L7, L9, L16 is the 1st, 2nd, 3rd, 5th, 7th, 9th, 16th leaf counted downward from the top meristem, respectively. The lower the leaf is located, the more aged and senescent it is.

Moreover, we observed the phenotype of leaves in the same phyllotaxy in *AaEIN3*-ox, WT, and RNAi plants. In *AaEIN3*-ox plants, the point at the edge of the 5th leaf counted downward from the top meristems (L5) began to show slight etiolation, and the etiolation phenomenon became more and more visible in the 7th, 9th, and 16th leaves counted downward from the top meristems (L7, L9, L16). Compared to *AaEIN3*-ox plants, L5 in WT plants has not shown etiolation, and the point at the edge of L7 and L9 in WT only exhibited slight trace of etiolation. The etiolation signs got more visible in L16 of WT plants, but the etiolation extent of L16 in WT plants was still lesser than that in *AaEIN3*-ox plants. On the other hand, none of the leaves L1–L16 in RNAi plants exhibited etiolation signs (**Figure 6**). These phenomena indicated *AaEIN3* overexpression accelerated leaf senescence, and repression of *AaEIN3* expression by RNAi delayed leaf senescence, which further demonstrated *AaEIN3* is a senescence-associated gene that can induce leaf senescence in *Artemisia annua*.

**FIGURE 7:**
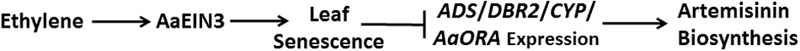
Illustration of the mechanism by which ethylene signaling negatively regulates artemisinin biosynthesis.

Taken together, our study proposed a mechanism by which ethylene negatively regulates artemisinin biosynthesis, shown in **Figure 7**. Ethylene signal promotes the senescence process of leaves in *Artemisia annua* via the mediation of AaEIN3, a key component in ethylene signaling pathway. And leaf senescence attenuates the expression of *ADS, DBR2, CYP71AV1*, and *AaORA*, thus reducing DHAA biosynthesis and finally leading to the decrease of artemisinin accumulation in *Artemisia annua*. Our work discovered the first negative regulator (AaEIN3) for artemisinin biosynthesis and provided more novel knowledge and clues on how plant hormone signals regulate artemisinin metabolic pathway. However, it’s still to be explored and studied as for the issues how the process of maturation and senescence of leaves modulates the expression of key genes involved in artemisinin biosynthesis, and what transcription factors or other signaling pathways get involved in this modulation. All of these issues need further research.

## Author Contributions

YT, LL, and KT conceived and designed the experiments. YT, TY, and PS performed the experiments. YT, XF, and QS analyzed the data. YT, XS, and KT contributed reagents, materials, and analysis tools. YT and KT wrote the manuscript.

## Conflict of Interest Statement

The authors declare that the research was conducted in the absence of any commercial or financial relationships that could be construed as a potential conflict of interest.
