# Supplementary material for: AaEIN3 Mediates the Downregulation of Artemisinin Biosynthesis by Ethylene Signaling Through Promoting Leaf Senescence in Artemisia annua
Source: Front Plant Sci. 2018 Apr 5;9:413. doi: 10.3389/fpls.2018.00413 (PMC5895717; doi:10.3389/fpls.2018.00413)
Supplement: Supplementary file 1 [file Table_1.DOCX]

Table S1 Primers used for qPCR analysis

| Primer Name | Primer Sequence (5’-3’) |
| --- | --- |
| AaEIN3-RT1 | CCACCAGAAGCAAAAATTTCCCATC |
| AaEIN3-RT2 | TGATTGGTTGGAAGGGATGTTTGAC |
| AaNAC2-RT1 | AGGGTATTGGAAGGCTACAGGG |
| AaNAC2-RT2 | TTGGGAGATTATTCGTCGCTAA |
| AaORA-RT1 | ATTTCCAACTAAACACGGTTGAGCCT |
| AaORA-RT2 | GGATCTTGAAGTGTTGCATATAATGAAAG |
| AaSAG12-RT1 | GGAGAAAGAAAGGTGCGGTAAC |
| AaSAG12-RT2 | CCATAAGACCACCCTCACATCC |
| Actin-RT1 | CCAGGCTGTTCAGTCTCTGTAT |
| Actin-RT2 | CGCTCGGTAAGGATCTTCATCA |
| ADS-RT1 | AATGGGCAAATGAGGGACAC |
| ADS-RT2 | TTTCAAGGCTCGATGAACTATG |
| CYP-RT1 | CACCCTCCACTACCCTTG |
| CYP-RT2 | GACACATCCTTCTCCCAGC |
| DBR2-RT1 | CTTGGGTTACAAGCTGTGGCTCAAG |
| DBR2-RT2 | ATATAATCAAAACTAGAGGAGTGACC |
